# Precipitation deficits increase high diurnal temperature range extremes

**DOI:** 10.1038/srep12004

**Published:** 2015-07-08

**Authors:** Bin He, Ling Huang, Qianfeng Wang

**Affiliations:** 1 College of Global Change and Earth System Science, Beijing Normal University, Beijing 100875, China; 2 Joint Center for Global Change Studies, Beijing 100875, China,; 3 College of Environment and Resources, Fuzhou University, Fuzhou 350108, China; 4 Academy of Disaster Reduction and Emergency Management, Beijing Normal University, Beijing 100875, China

## Abstract

The relationship between precipitation deficits and extreme hot temperatures has been documented in observation and modeling studies. However, it is unclear whether and how increases in maximum temperatures will impact diurnal temperature range (DTR) extremes. Here, we used observational data sets from meteorological stations in China to examine the trends in high DTR extremes from 1971 to 2013, represented by the percentage of high DTR days (%HDD) and maximum high DTR duration (MHDD), as well as their relationships with precipitation deficits over the past four decades in China. We identified both positive and negative trends in the %HDD and MHDD in China during each season, implying an inhomogeneous behavior of DTR and DTR extremes. Furthermore, we observed a significant negative relationship between precipitation deficits and the %HDD and MHDD during each season, and the relationship was strongest in the summer. The statistical analysis of this coupled behavior indicated that precipitation deficits were related to an increase in high DTR extremes, with a 22% average higher probability of the occurrence of DTR extremes after dry conditions than wet conditions in the summer. Knowledge from this study has important implications for interpreting climate anomalies.

Diurnal temperature range (DTR) is an important indicator of climate change^1^, and its variations can have significant impacts on public health^2^, agricultural productivity^3^, the carbon cycle in terrestrial ecosystems^4^,^5^, etc. For example, a large DTR could expose human communities to a high risk of a number of diseases^2^. Numerous studies have confirmed that a high DTR is a potential trigger for human mortality^6^,^7^. Hence, improvements in the ability to predict DTR abnormities are crucial for public management in many areas.

Changes in precipitation impact minimum temperatures (Tmin), maximum temperatures (Tmax), average temperatures (Tavg)^8^,^9^, and hence DTR trends^10^. Climate warming has significantly decreased the DTR over the past several decades^11^,^12^, but it is not yet known whether DTR extreme high values have been reduced. In addition, precipitation deficits are usually accompanied by high temperature extremes in summer^13^,^14^. The widely accepted explanation for this mechanism is that dry conditions favor more sunshine and less evaporative cooling^8^,^13^. To our knowledge, high temperature extremes are usually represented by indices based on daily maximum temperatures. Owing to the fact that small changes in maximum and minimum temperatures greatly impact DTR^11^, whether and to what extent precipitation deficits related to temperature abnormalities will impact DTR is still poorly understood. Hence, the goals of this study are to investigate the variations in DTR extremes and to explore the relationship between DTR extremes and precipitation deficits.

Many previous studies have focused on the long-term changes in DTR^11^,^12^, but few have investigated short-term abnormal DTR events. In China, some investigations have suggested the close relationship between DTR variation and public heath in several city of China^2^,^15^. For example, a study in Guangzhou indicated that 1°C increase in DTR was associated with 0.47% increase in total mortality^15^. So, it is of great importance to understand the variation of DTR extreme and related influencing factors in China.

In this study, we defined two indices to represent high DTR extremes: the percentage of high DTR days (%HDD) and maximum high DTR duration (MHDD) (see Methods). Based on observational data sets from meteorological stations in China from 1971 to 2013, the above two indices of DTR extremes were calculated and their trends were tested using linear regressions (see Methods). The standardized precipitation index (SPI) was employed to express dry conditions (see Methods). A simple correlation analysis was used to investigate the relationship between precipitation deficits and DTR extremes. In addition, quantile regression analysis was employed to examine the response of DTR extremes to precipitation deficits, which is crucial for exploring the plausible interaction mechanisms between these two variables^13^ (see Methods).

Figure 1 shows the linear trends in %HDD for each station from 1971–2013. Previous studies have agreed that the DTR has continuously decreased throughout China^16^,^17^, especially in winter; however, variations in seasonal high extreme DTR events are very complex, and both positive and negative trends exist. An obvious north–south division in the distribution of the %HDD trends was identified in the spring, with significant positive trends mainly observed in South China and significant negative trends mainly observed in North China. From spring to winter, the number of stations with significant positive trends gradually decreased, but the number of stations with significant negative trends increased and seemed to gradually expand from the northern to the eastern and southern parts of China. These results indicate that a continued decrease in DTR does not correspond to a similar decrease in DTR extreme events. Considering its potential impacts on public health, the change in the variability in DTR extreme events should be taken seriously. The linear trends in the DTR extremes indicated by the MHDD had a similar spatial distribution (Fig. S1).

Figure 2 shows the spatial distribution of the correlations between %HDD and SPI (standardized precipitation index) in different seasons. Dominant negative correlations were identified in all of the seasons, and the strongest relationships occurred in summer and autumn. The percentage of stations at which significant negative correlations (P < 0.05) were revealed was 53.6% in spring, 74.5% in summer, 69.3% in autumn, and 42.9% in winter. Obvious spatial differences could be identified for the distribution of the correlations. In spring and winter, strong negative relationships occurred in the southeastern and western areas of China. In summer, almost all areas exhibited a strong negative correlation, whereas in autumn, these areas decreased but were still extensively distributed. This strong coupling suggests that dry conditions (SPI < 0) were usually accompanied by increased DTR high extremes, and wet conditions (SPI > 0) were usually accompanied by decreased DTR high extremes. A negative relationship also existed between the MHDD and SPI, but the number of stations with significant MHDD and SPI correlations was less than those with significant %HDD and SPI correlations (Fig. S2), suggesting a higher influence of SPI on %HDD than on MHDD. The reason for this phenomenon is that the %HDD better describes the frequency of high DTR extremes, whereas the MHDD better represents the duration of high DTR extremes. High %HDD does not necessarily indicate high MHDD, but high MHDD usually occurs along with high %HDD.

To investigate whether the preceding precipitation deficits impacted the DTR extremes, correlations between the monthly %HDD and MHDD and the monthly SPI and preceding 1- and 2-month SPI were compared. As shown in Figs S3, S4 and S5, widely distributed negative correlations between these monthly values were identified, illustrating that 1) an inverse relationship between precipitation deficits and DTR extremes existed each month, and 2) preceding precipitation deficits also had strong effects on DTR extremes. The number of stations with significant correlations between the monthly %HDD (and MHDD) and the preceding 1- and 2-month SPI was less than that with significant correlations between the monthly %HDD (and MHDD) and SPI; in addition, the preceding 1-month precipitation seemed to have a greater impact on DTR extremes than the preceding 2-month precipitation, indicating stronger impacts on DTR extremes from short-term rather than long-term precipitati.on deficits.

Quantile regression allowed us to examine whether the effects of the SPI differed across quantiles of the %HDD conditional distributions. To investigate the overall conditions in China, the %HDD and SPI values from all the stations were included in the quantile regressions. Figure 3 presents scatter plots of the seasonal %HDD versus SPI values. Distinctive negative slopes representing the four seasons were identified in each of the four different quantiles. Moreover, the coefficients of the slopes gradually increased towards higher %HDD quantiles, indicating that dry conditions may have impacted a wide range of %HDD values, especially the top-tail of the conditional distribution. That is, high DTR extremes usually occurred with dry conditions. In addition, larger quantile regression line slopes were identified in summer, indicating stronger coupling between the %HDD and SPI in this season.

Quantile regressions of %HDD and SPI were also performed for each station in summer, as shown in Fig. S6. The spatially consistent negative slopes of the regression lines in the four different quantiles imply that the conditional distributions of the response of %HDD to SPI followed the same pattern. In addition, at some stations in northeastern, southwestern, and central China, a gradual increase in the magnitude of the negative coefficients of the quantiles illustrates a strong inverse coupling between dry conditions and higher DTR extremes in these regions. The results of the quantile regressions of MHDD and SPI are shown in Fig. S7; these results present a similar pattern to those in Fig. S6.

To quantify the strength of the relationship between DTR extremes and dry conditions, the frequency of high DTR events occurring after dry conditions (SPI < −0.8) and wet conditions (SPI > 0.8) were calculated, as shown in Fig. 4 and S8. For each station, the frequency was represented by the percentage of high DTR extremes (above-average %HDD and MHDD) that occurred in dry years (SPI < −0.8) or wet years (SPI > 0.8) from 1971 to 2013. Throughout China, the number of DTR extremes that occurred during dry conditions was consistently higher than that during wet conditions across the four seasons. The largest differences occurred in summer, with 22% of the DTR extremes occurring after dry conditions based on %HDD, and 17% based on MHDD (Table S1). For the majority of stations, the occurrence of high DTR extremes (%HDD) after dry conditions was >40% in the summer, but the occurrence after wet conditions was <20%. Table S2 shows the probability of dry conditions (or wet conditions) when high DTR extremes occurred. DTR extremes were found in a higher proportion of dry than wet years, especially in the summer. On the basis of the %HDD in the summer, DTR extremes occurred in ~33% of dry years and ~11% of wet years; based on the MHDD, the corresponding percentages were 29% and 13%, respectively. These results further confirm the coupling between DTR extremes and dry conditions.

Previous observations have indicated that the area-averaged DTR has decreased over land over the past several decades because of asymmetric temperature changes, with larger increases in the daily Tmin than Tmax^11^,^12^. According to modeling studies, negative trends in DTR are projected to persist in the future^1^. In China, the decreasing trend of DTR was also dramatic^18^. However, according to our findings, this persistent reduction in DTR does not correspond to a decrease in the high DTR extremes in some regions of China. Because of the comprehensive impacts of DTR anomalies on natural and social-economic systems, the variation in DTR and DTR extremes needs to be further examined on a larger spatial scale.

Another finding of this study is the coupling between precipitation deficits and DTR extremes. To explore the potential mechanisms responsible for the close relationship between high DTR extremes and dry conditions, we must understand how precipitation deficits will affect daily maximum and minimum temperatures. In light of previous knowledge (as noted in the introduction), a generally accepted physical mechanism that explains the interaction of precipitation anomalies and temperature variations is soil moisture anomalies, which affect the energy budget balance^8^,^13^,^14^. The heat budget influences surface temperature by regulating the ratio of sensible heat flux to latent heat flux (i.e., the Bowen ratio)^8^. Precipitation deficits cause low soil moisture availability, which constrains evaporative cooling, causing surface air to be heated by the sensible heat flux. That is to say, dry conditions corresponding to higher Bowen ratios are responsible for increased temperatures. In addition, the lack of cloud cover on dry days further strengthens this heating process^8^. During wet conditions, the reverse process occurs; a larger amount of the available energy is used to increase evaporation, which is supported by increased soil moisture. This is the reason most of the land surface is usually warm in dry years but cold in wet years^19^. Previous observation and modeling studies have suggested that dry conditions trigger an increase in maximum temperatures, and hence cause more high temperature extremes^13^,^14^,^20^.

The reduced moisture content of the atmosphere and ground soil caused by precipitation deficits can result in a decrease in the overnight cloud coverage, which can cause nighttime minimum temperatures to be slightly lower than normal^21^. Clouds can increase minimum temperatures by enhanced downward longwave radiation^21^. Without clouds to act as an insulator for nighttime temperatures, the surface should lose heat more efficiently. The above analysis suggests that dry conditions increase maximum temperatures but decrease minimum temperatures, and hence enlarge the DTR. This is a simple explanation of our findings, and many other factors, such as atmospheric circulation^22^, land surface conditions^23^, humidity^24^, greenhouse gases, and aerosols^10^, jointly compose a very complex process. Therefore, a more in-depth study that combines observations and models is needed.

Currently, many studies are focused on the relationships between different climate variables, but few involve future climate projections^24^. Knowledge from this study can be used to interpret climate anomalies occurred in China, which have potential implications for model-based analyses and projections of climate extremes. This study also suggests that the interaction between precipitation deficits and air temperature is more complex, and its impacts are more comprehensive than previously expected.

## Methods

### Observed climate data

The observed daily precipitation and maximum and minimum temperature data used in this study was collected from the SURF_CLI_CHN_MUL_DAY_V3.0 dataset, which was downloaded from the China Meteorological Data Sharing Service System ( http://cdc.nmic.cn/home.do). This dataset contains daily measurements of eight meteorological factors (air pressure, temperature, precipitation, evaporation, etc^25^) from 824 stations from January 1951 to July 2014. According to the dataset information ( http://cdc.nmic.cn/datasets.do? dsid = SURF_CLI_CHN_MUL_DAY_3.0#), the full dataset was quality controlled and homogenized before its release. Details about the methods for raw data homogenizing could be found in Xu *et al.*’s^26^ study. The accuracy of this dataset was greatly improved than the raw data^26^, and it has been frequently used to detect temperate extremes in China^26^,^27^. Because of frequent data gaps in earlier years caused by instrument malfunctions^28^, we excluded the data prior to 1971. In addition, to ensure the reliability of our analysis, only stations with no missing daily temperature or precipitation data from 1971 to 2013 were included in the analysis. To retain the data as much as possible, we sorted out the station meeting our criteria season by season. Finally, 633, 658, 649, and 662 stations meeting this standard were selected for the spring, summer, autumn, and winter analyses, respectively.

### DTR extreme indices and trends tests

According to the recommendation of the Central and Eastern Europe Climate Change Impact and Vulnerability Assessment (see http://www.cecilia-eu.org/), we defined two percentile threshold indices to represent high DTR extremes: %HDD and MHDD. Percentile-based indices based on probability and statistics are commonly used to explore temperature extremes^29^. In contrast to threshold-based indices, percentile-based indices enable easier comparisons across different climatic regions^13^. In light of previous studies^13^,^14^,^30^,^31^, we applied an empirically derived 90th percentile threshold to the two indices. The %HDD is similar to a widely used index, the number of hot days per month (NHD)^13^,^14^, which indicates high temperature extremes. The %HDD was defined as the percentage of days per month or season in which the DTR exceeded the long-term 90th percentile. The %HDD was calculated from 215 values (5 per year for 43 years) based on 5-consecutive-day moving windows centered on each calendar day and the 90th percentile thresholds from 1971–2013. For example, to determine the 90th percentile of the DTR that was used to evaluate whether the DTR at a station on July 15, 1981 was extreme, all the daily DTRs during the period from July 13 through July 17 for each year of the dataset (1971–2013) were ranked, and the 90th percentile of the ranked data was determined. The MHDD was defined as the maximum number of consecutive days in which the DTR exceeded the 90th reference-period percentile. The 90th percentile of the DTR used in the MHDD index was determined in the same way as that for the %HDD index. The MHDD was obtained by counting the maximum number of consecutive high DTR days per month or per season. Linear regressions were used to identify the trends in %HDD and MHDD at each station over the past four decades, and the 5% statistical significance level was discussed^32^.

### SPI

A commonly used drought index, the Standardized Precipitation Index (SPI), was employed here to express dry conditions. The SPI was calculated based solely on precipitation^33^, and thus excluded the interference from temperature differences in dry and wet condition analyses. Another advantage of the SPI is its multi-scale character, which allowed us to assess the precipitation conditions over different time periods. A detailed description of SPI and its computation can be found in Guttman’s paper^34^. In this study, 1-month, 2-month, and 3-month SPIs were calculated for each station to assess the dry conditions during different months and seasons.

### Correlation analysis and quantile regression

A simple correlation analysis was used to investigate the relationship between monthly and seasonal precipitation deficits and DTR extremes. In addition, we considered the impacts of preceding precipitation deficits on DTR extremes. For instance, the DTR extreme that occurred in July was influenced by the June or May–June precipitation deficits. The preceding precipitation deficits were represented by 1- and 2-month SPIs, which were calculated based on the total precipitation in June and May–June. The correlations between the monthly %HDD and MHDD values and the monthly SPI and preceding 1- and 2-month SPI values were compared. Only correlation coefficients with p < 0.05 were considered statistically significant and used in the analysis. Quantile regression is a robust way to estimate the conditional distributions of a response variable in a linear model, and it can provide a more complete view of possible causal links between variables compared to mean regressions^35^. Quantile regression has been widely used in ecology^35^, economics^36^, sociology^37^, etc. Recently, it had been frequently employed to examine the relationships between extreme high temperatures and soil moisture deficits^13^,^14^. Details about this method and the related formulas were described by Hirschi *et al.*^14^,^38^

## Additional Information

**How to cite this article**: He, B. *et al.* Precipitation deficits increase high diurnal temperature range extremes. *Sci. Rep.* **5**, 12004; doi: 10.1038/srep12004 (2015).

## Supplementary Material

Supplementary Information

## Figures and Tables

**Figure 1:**
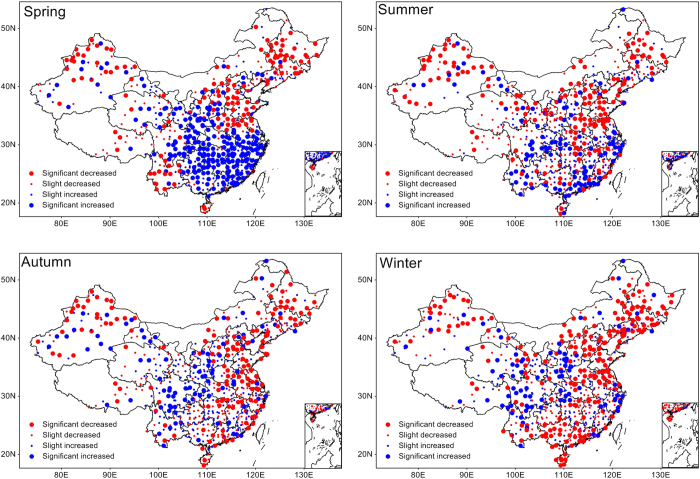
Trends tests. Linear trends in %HDD for different seasons from 1971–2013. The %HDD was calculated from 215 values (5 × 43yr) based on 5-consecutive-day moving windows centered on each calendar day and 90th percentile thresholds from 1971–2013. The linear trend in %HDD was examined with linear regressions. Slopes were considered significant for p < 0.05. Red dots indicate negative trends, and blue dots indicate positive trends. The map was generated with MeteoInfo 1.1.3 (http://www.meteothinker.com/).

**Figure 2:**
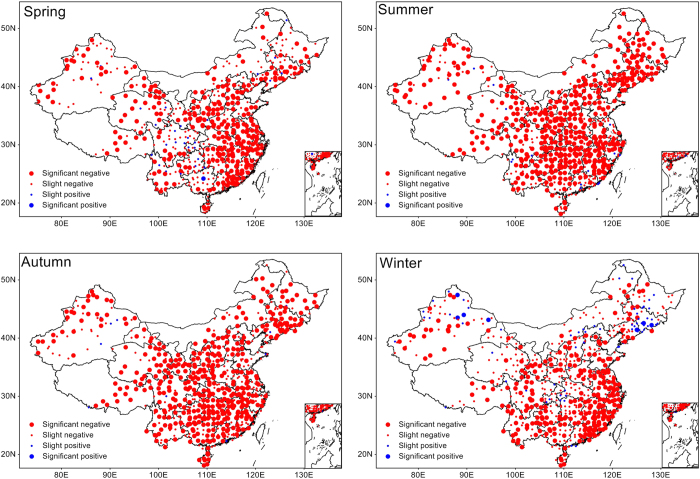
Correlation analysis. Correlation between precipitation deficits (SPI) and DTR extremes (%HDD) for different seasons during 1971–2013. The correlation coefficients were calculated by using Person’s correlation analysis. Correlations were considered significant for p < 0.05. Red dots indicate negative relationships, and blue dots indicate positive relationships. The map was generated with MeteoInfo 1.1.3 ( http://www.meteothinker.com/).

**Figure 3:**
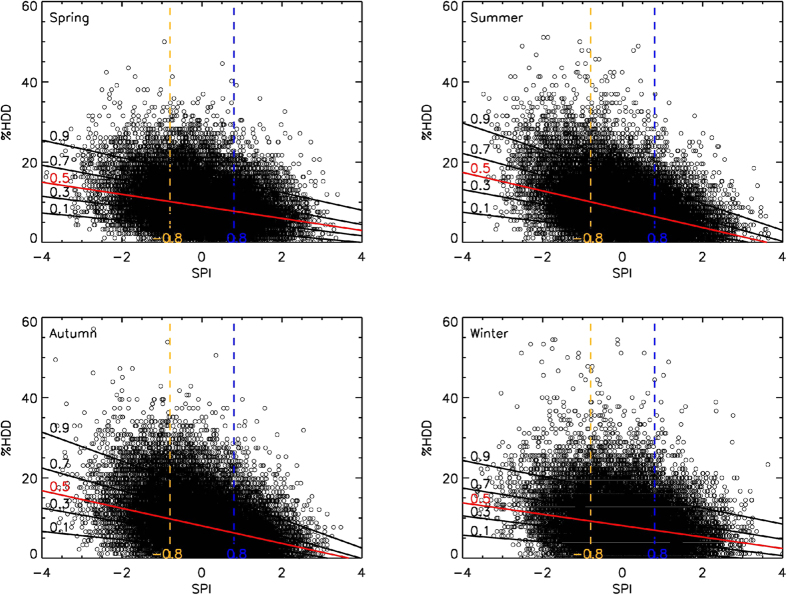
Quantiles analysis. Scatter plots of seasonal %HDD and SPI for all stations in China from 1971–2013. %HDD and SPI values from all stations during 1971–2013 were included in the quantile regressions. Quantiles selected as regression lines are 0.1, 0.3, median (0.5, red line), 0.7, and 0.9, respectively. Dry conditions were defined as SPI < −0.8 (yellow lines), and wet conditions were defined as SPI > 0.8 (blue lines). The map was generated with IDL 8.1.

**Figure 4:**
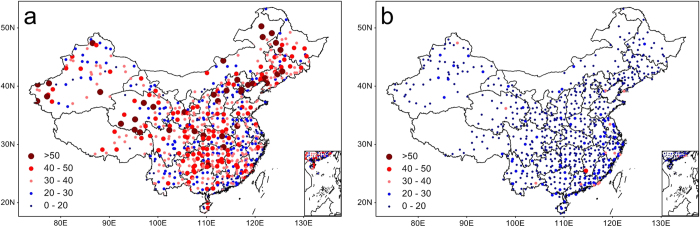
Occurrence probability of DTR extremes. Spatial distributions of the occurrence probability of DTR extremes occurring after dry conditions (SPI < −0.8) (**a**) and wet conditions (SPI > 0.8) (**b**) in the summer. The occurrence frequency was represented by the percentage of high DTR extremes (above-average %HDD) that occurred in dry years (SPI < −0.8) or wet years (SPI > 0.8) during 1971–2013. The map was generated with MeteoInfo 1.1.3 ( http://www.meteothinker.com/).
