# Supplementary material for: Precipitation deficits increase high diurnal temperature range extremes
Source: Sci Rep. 2015 Jul 8;5:12004. doi: 10.1038/srep12004 (PMC4495382; doi:10.1038/srep12004)
Supplement: Supplementary Information [file srep12004-s1.doc]

**Supplementary Information**

**Precipitation deficits increased high diurnal temperature range extremes**

**Bin He**^1,2^, **Ling Huang**^1,2^, **Qianfeng Wang**^3^

 ^1^College of Global Change and Earth System Science, Beijing Normal University, Beijing 100875, China,^2^Joint Center for Global Change Studies, Beijing 100875, China,^3^Academy of Disaster Reduction and Emergency Management，Beijing Normal University, Beijing 100875, China.

Correspondence and requests for materials should be addressed to B.H.([hebin@bnu.edu.cn](mailto:hebin@bnu.edu.cn))

**
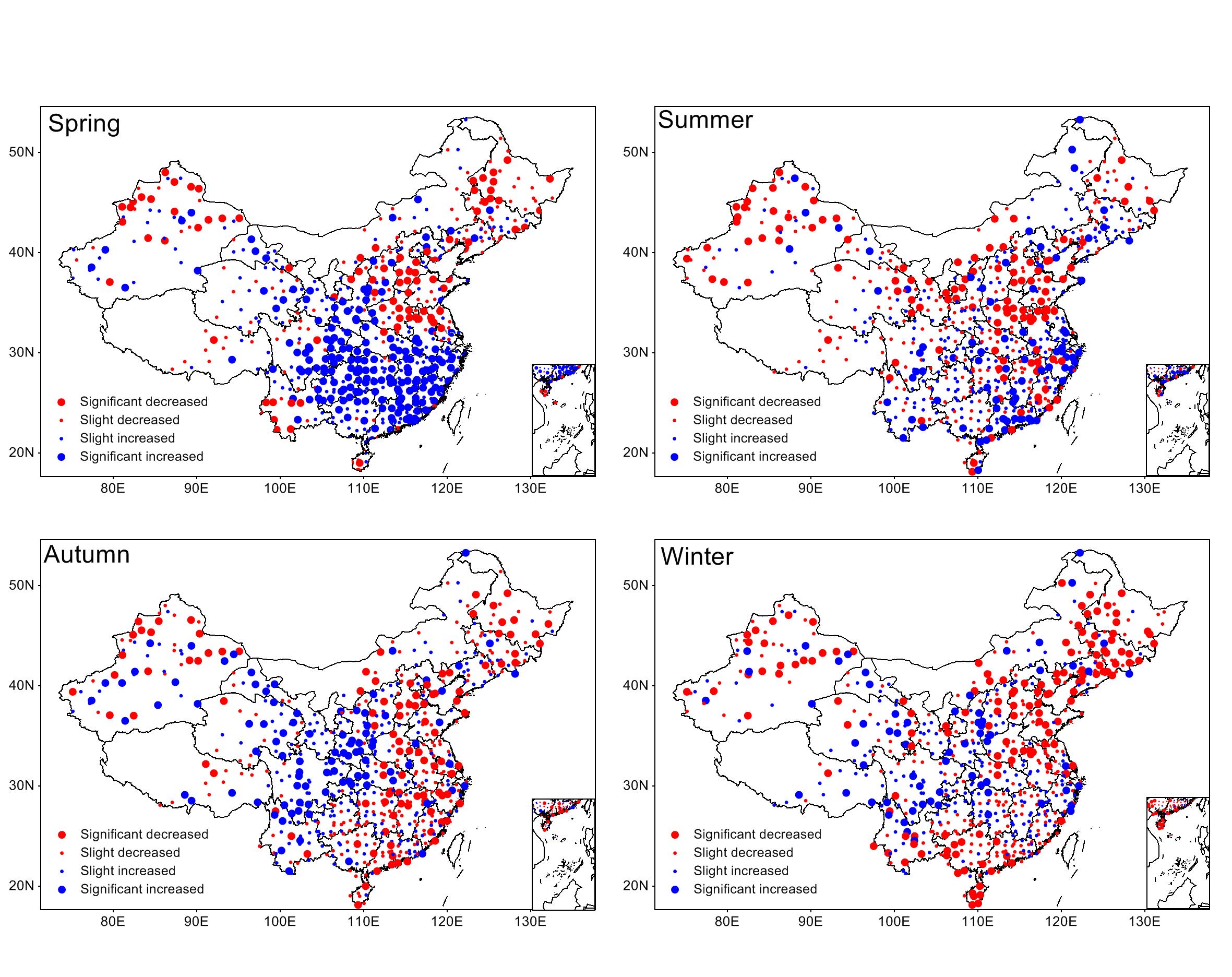
**

**Supplementary Figure 1: Trends tests.** Linear trends in MHDD for different seasons from 1971–2013. The MHDD was obtained by counting the maximum number of consecutive high DTR days per season. The high DTR was calculated from 215 values (5 × 43 yr) based on 5-consecutive-day moving windows centered on each calendar day and 90th percentile thresholds from 1971–2013. The linear trend in MHDD was examined with linear regressions. Slopes were considered significant for p < 0.05. Red dots indicate negative trends, and blue dots indicate positive trends. The map was generated with MeteoInfo 1.1.3 (<http://www.meteothinker.com/>).


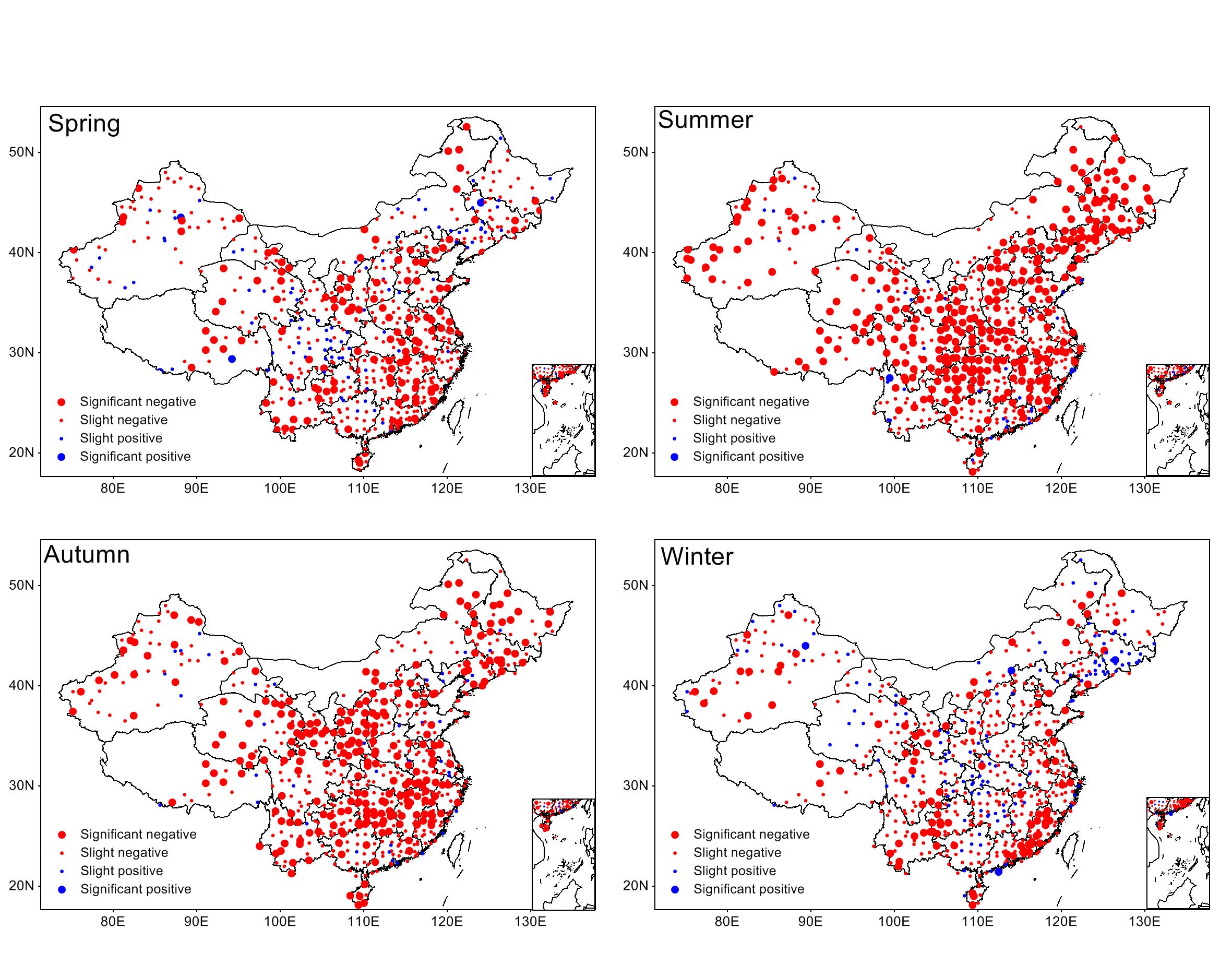


**Supplementary Figure 2: Correlation analysis.** Correlations between precipitation deficits (SPI) and DTR extremes (MHDD) for different seasons during 1971–2013. The correlation coefficients were calculated by using Person’s correlation analysis. Correlations were considered significant for p < 0.05. Red dots indicate negative relationships, and blue dots indicate positive relationships. The map was generated with MeteoInfo 1.1.3 (<http://www.meteothinker.com/>).

**Monthly %HDD VS SPI**


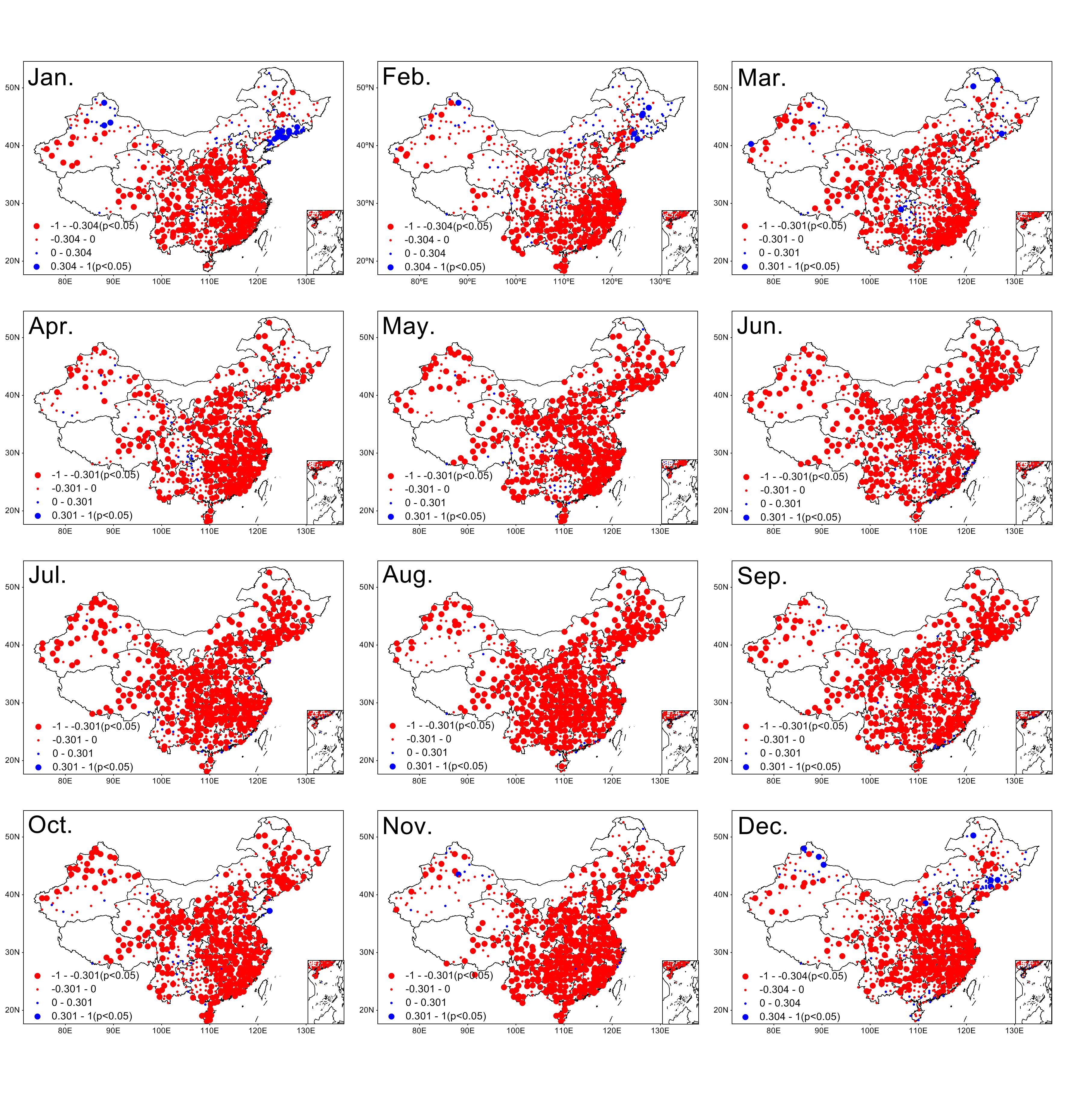


**Monthly MHDD VS SPI**

**
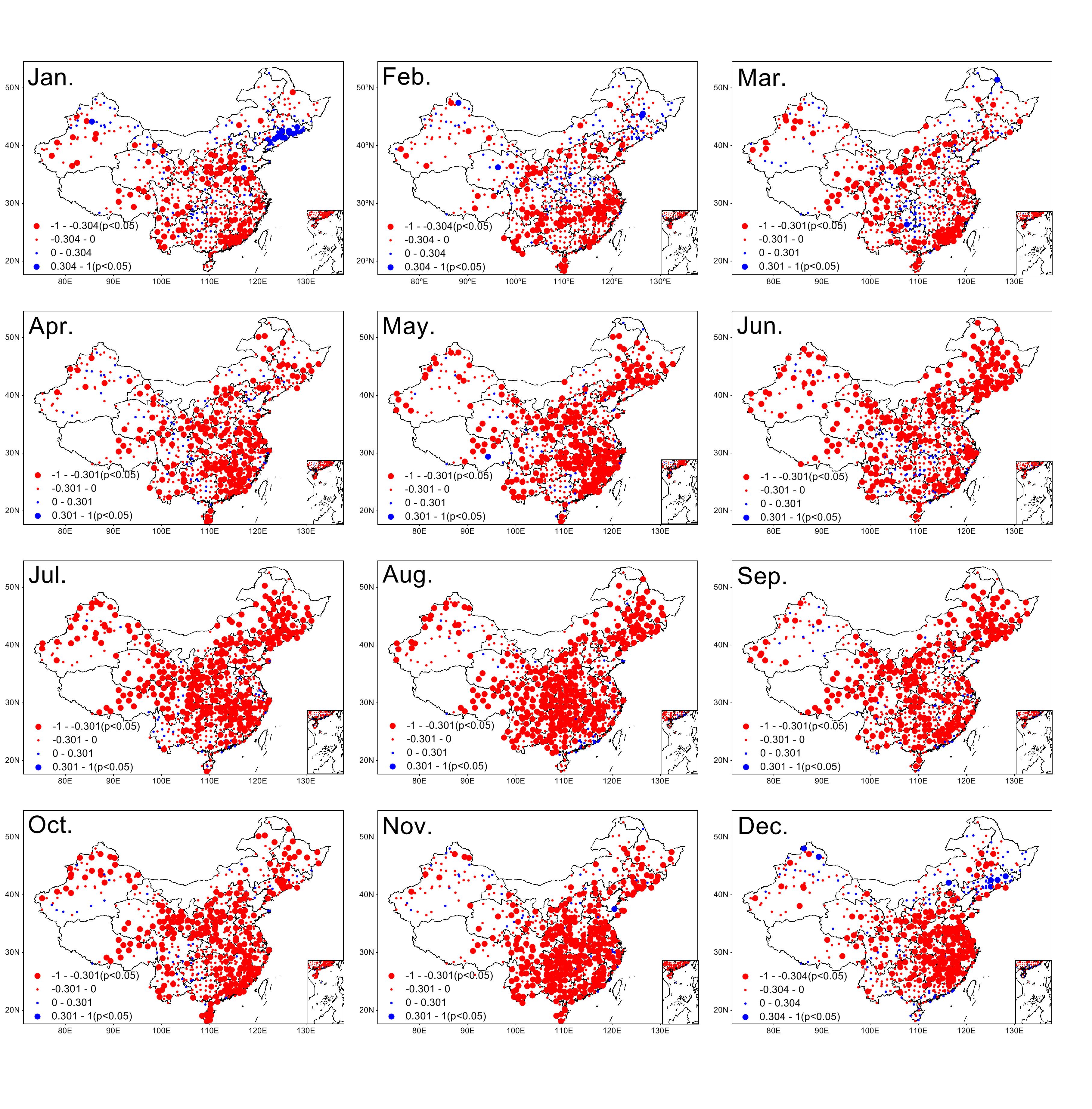
**

**Supplementary Figure 3:** Correlation between DTR extremes (%HDD and MHDD) and precipitation deficits (SPI) of the same month from 1971-2013. The correlation coefficients were calculated by using Person’s correlation analysis. Correlations were considered significant for p < 0.05. Red dots indicate negative relationships, and blue dots indicate positive relationships. The map was generated with MeteoInfo 1.1.3 (<http://www.meteothinker.com/>).

**Monthly %HDD VS Preceding 1-month SPI**


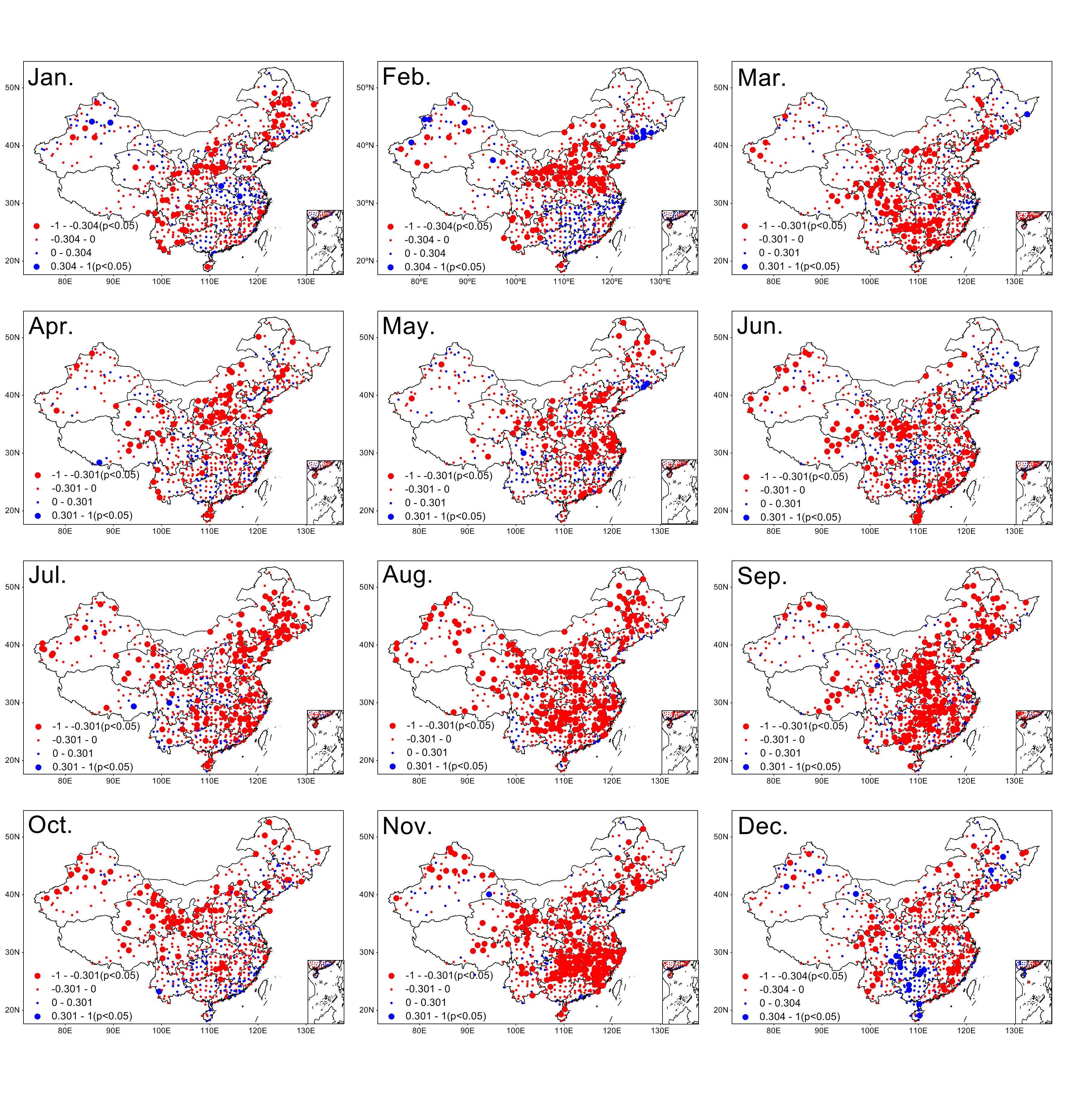


**Monthly MHDD VS Preceding 1-month SPI**

**
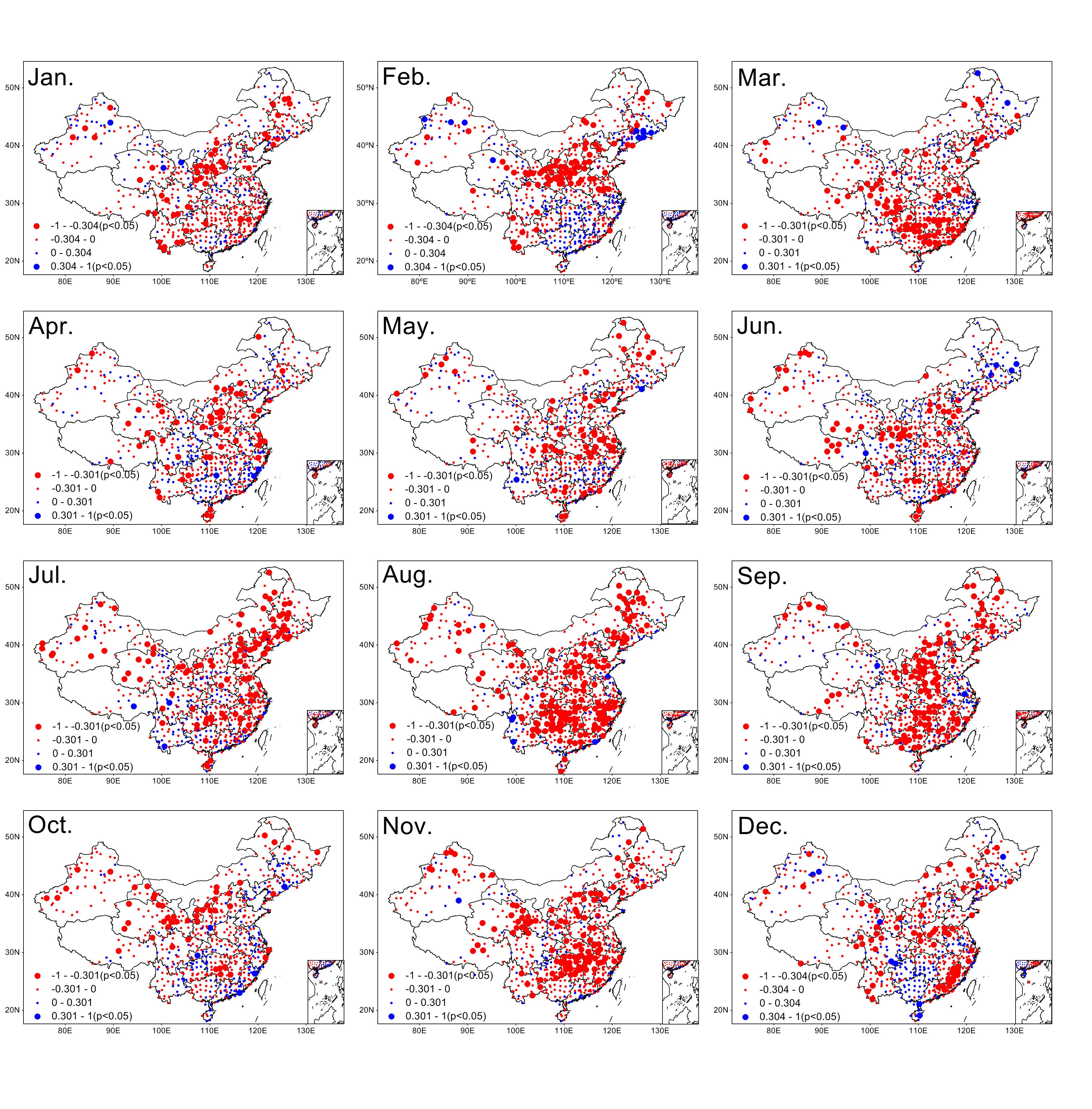
**

**Supplementary Figure 4:** Correlation between monthly DTR extremes (%HDD and MHDD) and precipitation deficits (SPI) of the preceding 1-month from 1971-2013. The correlation coefficients were calculated by using Person’s correlation analysis. Correlations were considered significant for p < 0.05. Red dots indicate negative relationships, and blue dots indicate positive relationships. The map was generated with MeteoInfo 1.1.3 (<http://www.meteothinker.com/>).

**Monthly %HDD VS Preceding 2-month SPI**


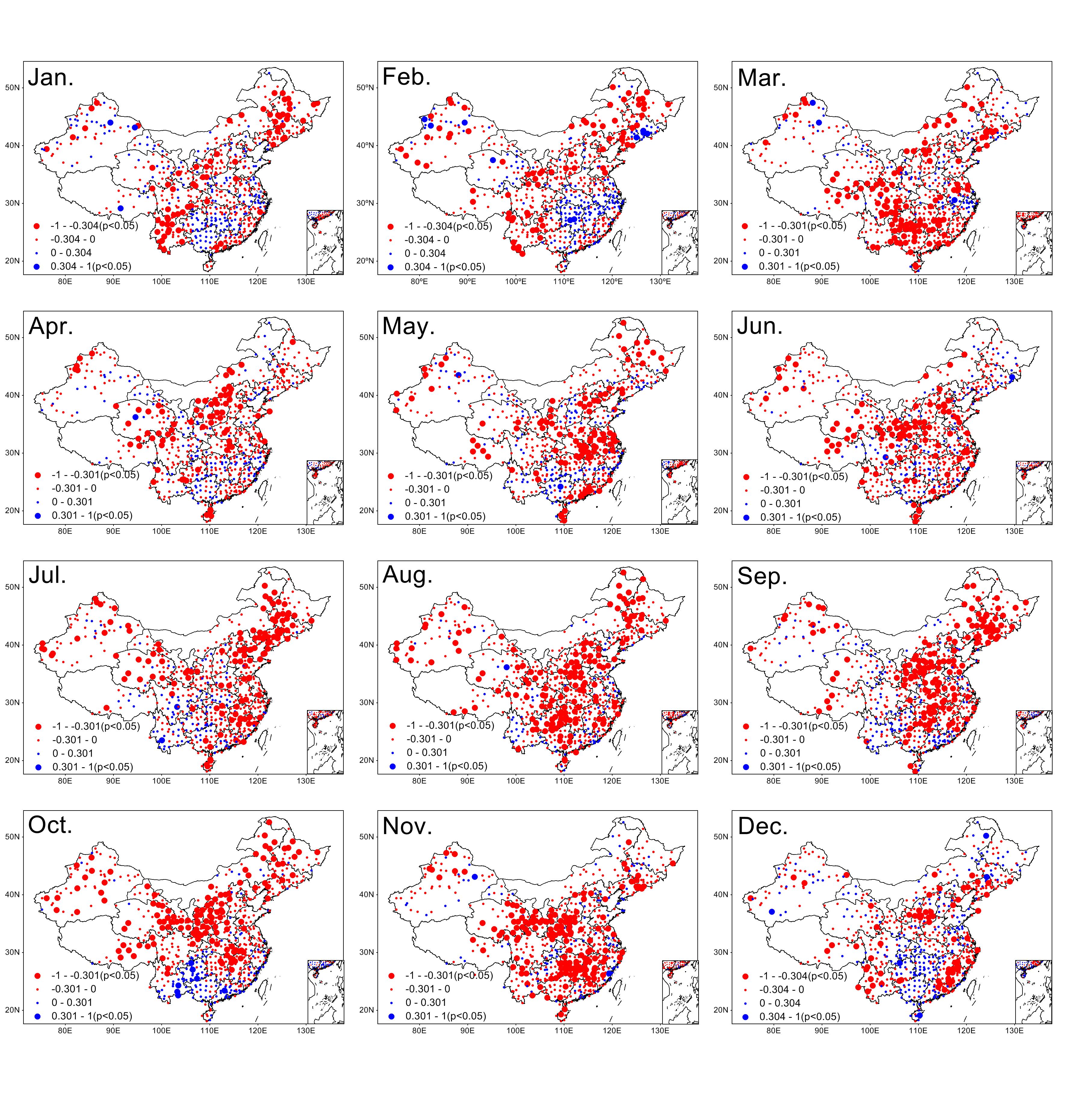


**Monthly MHDD VS Preceding 2-month SPI**


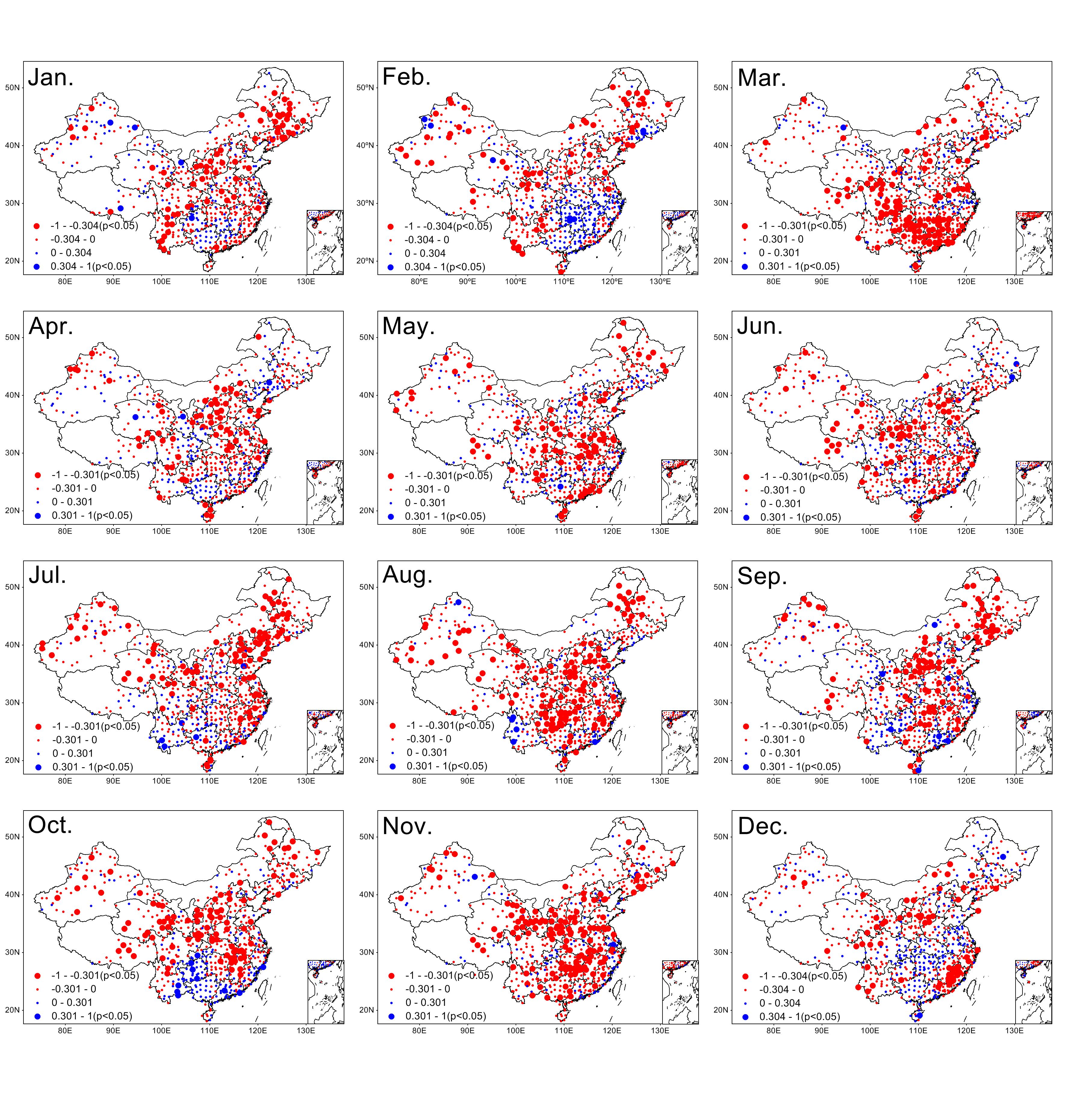
**Supplementary Figure 5:** Correlation between monthly DTR extremes (%HDD and MHDD) and precipitation deficits (SPI) of the preceding 2-month from 1971-2013. The correlation coefficients were calculated by using Person’s correlation analysis. Correlations were considered significant for p < 0.05. Red dots indicate negative relationships, and blue dots indicate positive relationships. The map was generated with MeteoInfo 1.1.3 (<http://www.meteothinker.com/>).


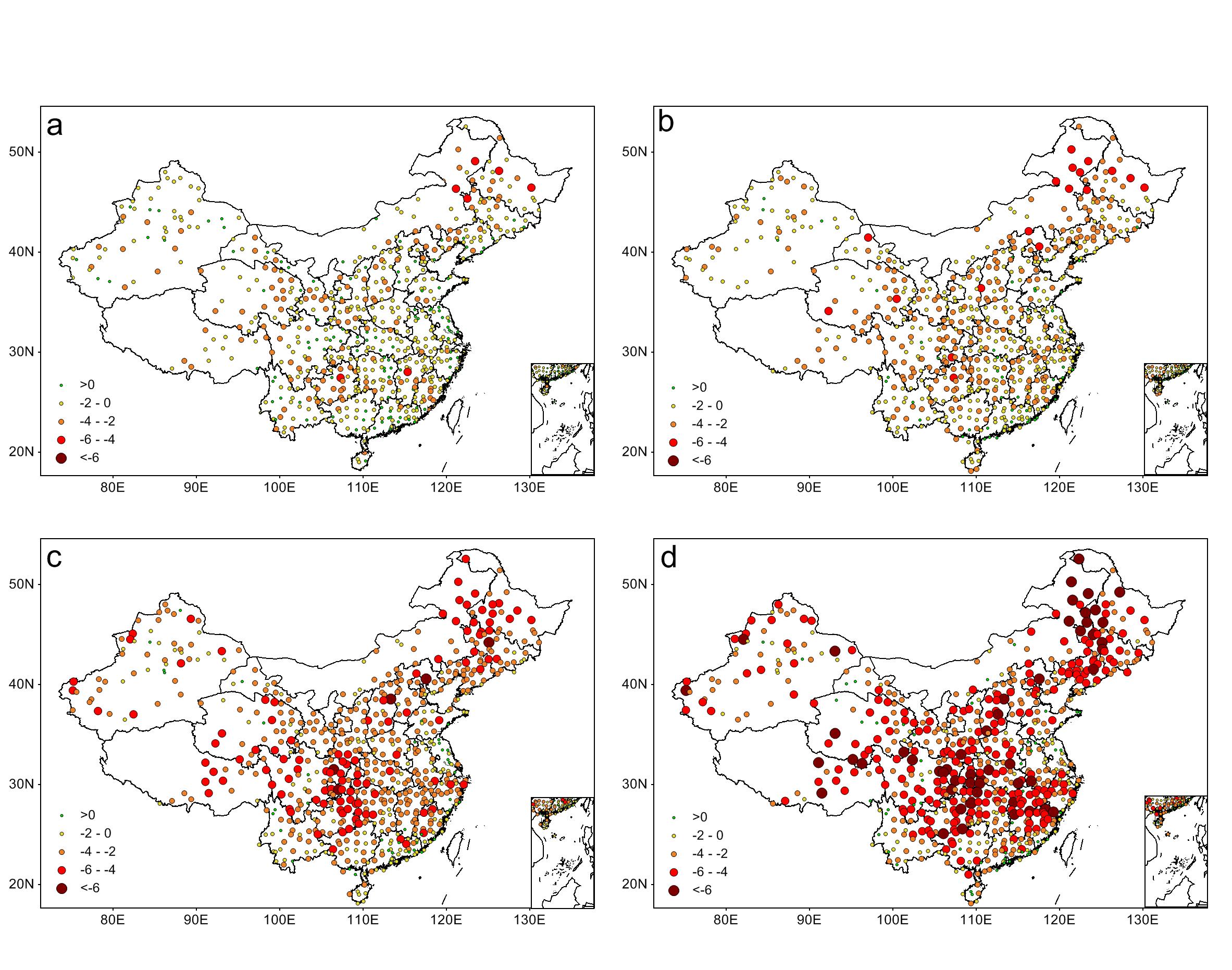


**Supplementary Figure 6:** Quantile regressions of %HDD and SPI for each station in summer for 1971-2013. The slope of regression lines for a selection of distinct quantiles are 10th(a), 30th(b), 70th(b) and 90th(d). Red dots indicate negative slopes. The map was generated with MeteoInfo 1.1.3 (<http://www.meteothinker.com/>).


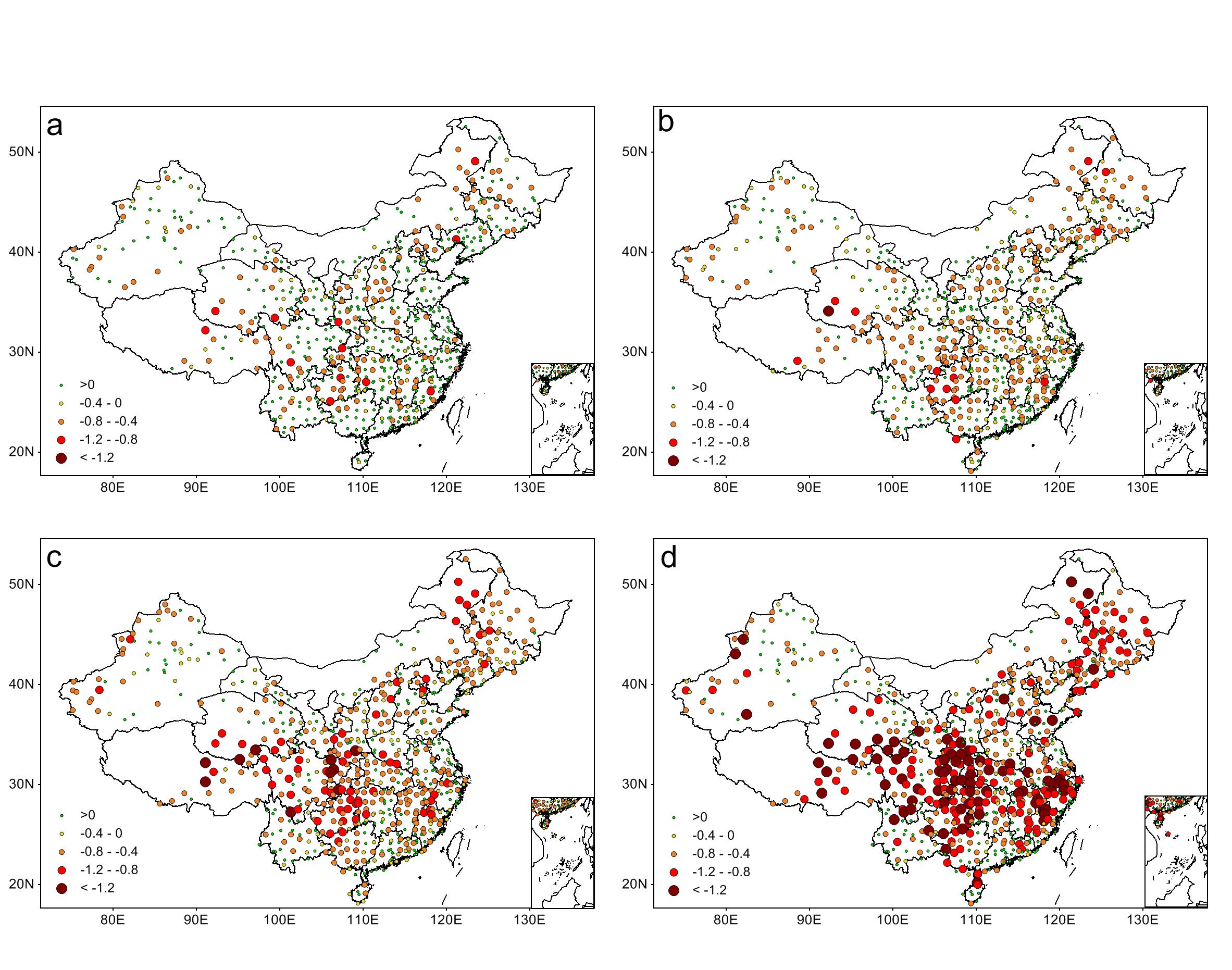


**Supplementary Figure 7:** Quantile regressions of MHDD and SPI for each station in summer for 1971-2013. The slope of regression lines for a selection of distinct quantiles are 10th(a), 30th(b), 70th(b) and 90th(d). Red dots indicate negative slopes.The map was generated with MeteoInfo 1.1.3 (<http://www.meteothinker.com/>).


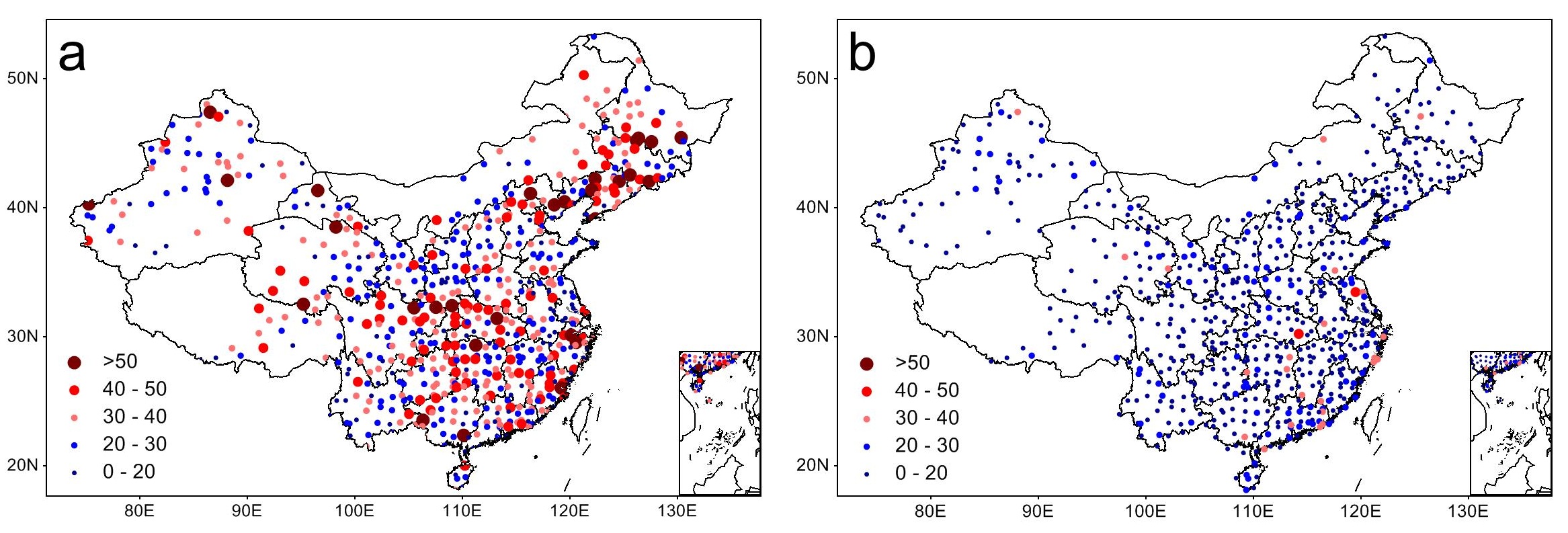


**Supplementary Figure 8:** Spatial distributions of occurrence probability of DTR extremes (MHDD) happened after dry conditions (SPI ＜ -0.8) (a) and wet conditions (SPI ＞ 0.8) (b) in the summer. The occurrence frequency was represented by the percentage of high DTR extremes (above-average MHDD) that occurred in dry years (SPI < −0.8) or wet years (SPI > 0.8) during 1971–2013. The map was generated with MeteoInfo 1.1.3 (<http://www.meteothinker.com/>).

**Supplementary Table 1:** Occurrence probability of DTR extremes that happened after dry (SPI ＜ -0.8) and wet conditions (SPI ＞ 0.8) for different season

| **Seasons** | **%HDD (%)** | | **MHDD (%)** | |
| --- | --- | --- | --- | --- |
|  | Dry  (SPI ＜ -0.8) | Wet  (SPI ＞ 0.8) | Dry  (SPI ＜ -0.8) | Wet  (SPI ＞ 0.8) |
| Spring | 29.18 | 13.68 | 26.09 | 16.44 |
| Summer | 33.22 | 11.13 | 30.40 | 13.27 |
| Autumn | 32.77 | 10.50 | 28.74 | 13.98 |
| Winter | 27.66 | 14.65 | 25.03 | 17.49 |

**Supplementary Table 2:** Probability of a soil condition being dry (SPI ＜ -0.8) or wet (SPI ＞ 0.8) when a high DTR extreme occurs

| **Seasons** | **%HDD (%)** | | **MHDD (%)** | |
| --- | --- | --- | --- | --- |
|  | %HDD dry | %HDD wet | MHDD dry | MHDD wet |
| Spring | 30.43 | 14.36 | 26.98 | 17.55 |
| Summer | 33.14 | 11.33 | 29.02 | 13.25 |
| Autumn | 32.32 | 10.70 | 27.84 | 14.33 |
| Winter | 28.57 | 15.47 | 25.28 | 18.50 |
